# Refined Carbon Emission Measurement Based on NPP-VIIRS Nighttime Light Data: A Case Study of the Pearl River Delta Region, China

**DOI:** 10.3390/s23010191

**Published:** 2022-12-24

**Authors:** Jian Yang, Weihong Li, Jieying Chen, Caige Sun

**Affiliations:** 1 School of Geography, South China Normal University, Guangzhou 510631, China; 2 SCNU Qingyuan Institute of Science and Technology Innovation Co., Ltd., Qingyuan 511517, China

**Keywords:** Pearl River Delta, night light data, CO_2_ emissions, optimized regression model, spatial pattern

## Abstract

The accurate measurement of CO_2_ emissions is helpful for realizing the goals of “carbon neutralization” and “carbon peak”. However, most current research on CO_2_ emission measurements utilizes the traditional energy balance coefficient and top-down methods. The data granularity is large, and most studies are concentrated at the national, provincial, municipal, or district/county administrative unit scale. As an important part of the Guangdong–Hong Kong–Macao Greater Bay Area of China, the Pearl River Delta region has good nighttime light vitality and faces huge carbon emission pressure. Using the Pearl River Delta as the research area, this study constructed an optimized pixel-scale regression model based on NPP-VIIRS (The Visible Infrared Imaging Radiometer Suite on the Suomi National Polar-Orbiting Partnership spacecraft) nighttime light data and CO_2_ emissions data at the district and county levels for 2017. In addition, the spatial pattern of CO_2_ emissions in the Pearl River Delta was analyzed based on the predicted CO_2_ emission status. The results showed that the spatial pattern of CO_2_ emissions in the Pearl River Delta had the distinct characteristics of the “center-edge” effect, the spatial spillover effect, and high-value aggregation, which should be considered when making related social or public decisions.

## 1. Introduction

Global warming has been widely recognized as a major issue that urgently needs to be alleviated, and it has been put on the agenda of every country globally [1]. Carbon emissions generated by the production processes, lifestyle, and operation of human society are among the main causes, and CO_2_ emissions are the main component of carbon emissions. China accounts for a significant proportion of global CO_2_ emissions and is the world’s largest carbon emitter [2,3,4]. To ensure China’s contribution to the fight against global warming, the Chinese government strives to attain the “carbon peak” by 2030 and “carbon neutrality” by 2060 so as to achieve green and low-carbon circular development. To meet this goal, the scientific and accurate measurement of CO_2_ emissions from the earth’s surface is of great significance to revealing the spatial pattern of CO_2_ emissions and providing an auxiliary and theoretical basis for the formulation of carbon emission policies in line with regional development.

Many scholars, at home and abroad, have attempted to use different methods to measure carbon emissions. From the perspective of research data, most research is based on energy statistics in statistical yearbooks of administrative units at all levels [5,6]. A multi-scale carbon emission estimation model for the Yellow River Basin was constructed based on the statistical carbon emission data of provincial energy consumption [7,8], and the evolution characteristics of carbon emissions from energy consumption were analyzed at multiple spatial and temporal scales [9]. Moreover, the sources of energy statistics are not completely unified, and finding carbon-related data sources remains challenging, making it difficult to compare the results. At the research scale level, owing to a lack of statistical data for municipal- and county-level administrative units, most of the existing studies are based on national and provincial scales [10]. Studies and analyses of carbon emissions at the municipal and county level [11] are scarce, and studies at the pixel level are rare. In terms of research methods, bottom-up methods, such as the carbon emission coefficient method, are mainly used [12], although they lack real-time carbon emission data. Fine and real-time measurement methods can further reflect the spatial and temporal distribution characteristics of carbon emissions scientifically and accurately and provide support for region-specific carbon emission policy guidance.

Nighttime light data have the advantages of a wide coverage, a long time span, and a simultaneous large area of ground information [13]. It can reveal the intensity of economic and human activities and has become one of the most important geographic information data [14,15,16,17]. With the rapid development of remote sensing technology, many scholars have increasingly utilized nighttime light data in various studies such as the multi-center extraction of urban agglomeration and the estimation of economic and social factors. Previous studies have demonstrated a correlation between nighttime light data and carbon emissions [13], which can be used to measure carbon emissions. However, there are relatively few studies on the application of nighttime light data in the field of carbon emissions. In general, most existing studies use DMSP-OLS (Defense Meteorological Satellite Program-Operational Linescan System) nighttime light data for measurement, which stopped updating after 2013, whereas NPP-VIIRS (The Visible Infrared Imaging Radiometer Suite on the Suomi National Polar-Orbiting Partnership spacecraft) nighttime light data not only filled the data gap after 2013 but also have a higher spatial resolution, which is more suitable for recent research.

Overall, the existing literature summarizes the traditional means of carbon emission measurement research, but the research scale is large, there are few pixel-level studies, and most of these take the administrative unit as the research object. It is difficult to determine the differences in the refined spatial distribution of carbon emissions. As the leading area of economic development in the Guangdong–Hong Kong–Macau Greater Bay Area, the Pearl River Delta region consumes considerable energy and is one of the key areas for controlling carbon emissions. Therefore, in this study, taking the Pearl River Delta region as the focal area and utilizing NPP-VIIRS nighttime lighting data and district- and county-level CO_2_ emission data, CO_2_ emission data were retrieved through the nighttime light index. An optimized pixel-scale regression model was constructed so that the spatial distribution unit of CO_2_ emissions was refined from the administrative unit scale to the pixel scale, realizing a fine simulation of the spatial distribution of carbon emissions and extracting the spatial distribution pattern of carbon emissions so as to provide a scientific basis for the reasonable control of carbon emissions. At the same time, it also provides a reference for the fine-grained carbon emission calculation research of the bay area and urban agglomeration.

## 2. Research Areas and Data Sources

### 2.1. Overview of the Study Area

**Figure 1:**
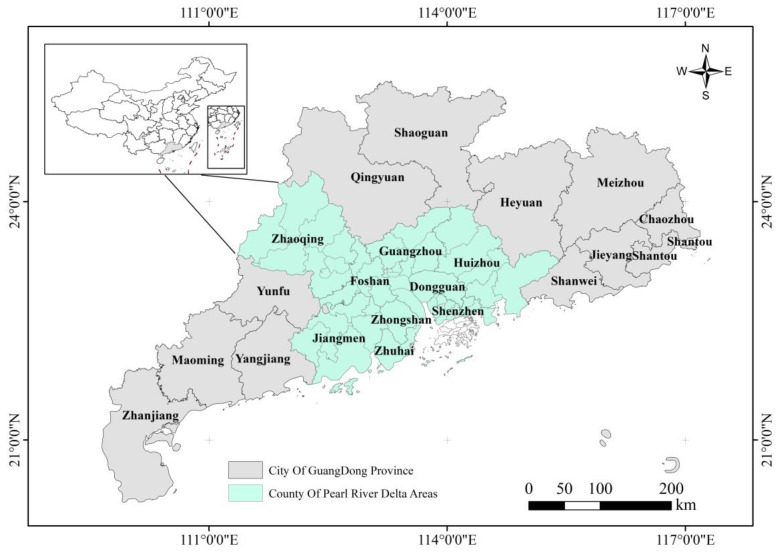
Sketch map of the study area.

The Pearl River Delta is located in the central and southern parts of Guangdong Province, connecting the two special administrative regions of Hong Kong and Macao to the south. It is the “south gate” of China, the core and prosperous home of Cantonese culture, and an important part of the Guangdong–Hong Kong–Macau Greater Bay Area. In 2017, the Pearl River Delta covered a total area of 55,368.7 km^2^, including Guangzhou, Jiangmen, Zhongshan, Zhuhai, Huizhou, Dongguan, Shenzhen, Zhaoqing, and Foshan (Figure 1), with a GDP(Gross Domestic Product) of CNY 7.58 trillion. Since the reforms and the opening up of the economy, the Pearl River Delta region has been one of the most economically dynamic regions in China, accounting for less than 1/3 of the area of Guangdong Province but attracting more than half of the province’s population and recording nearly 80% of the total economic output. It plays a prominent and strategic role in the overall situation of national economic and social development, reform, and opening up. It is also one of the largest urban agglomerations in the world, with a great driving force and potential for development. However, this immense driving force of development means that there will inevitably be an accompanying huge carbon emissions expenditure in the Pearl River Delta in the future. Therefore, studying fine measurements of CO_2_ emissions in the Pearl River Delta is of practical significance.

### 2.2. Data Source

**Figure 2:**
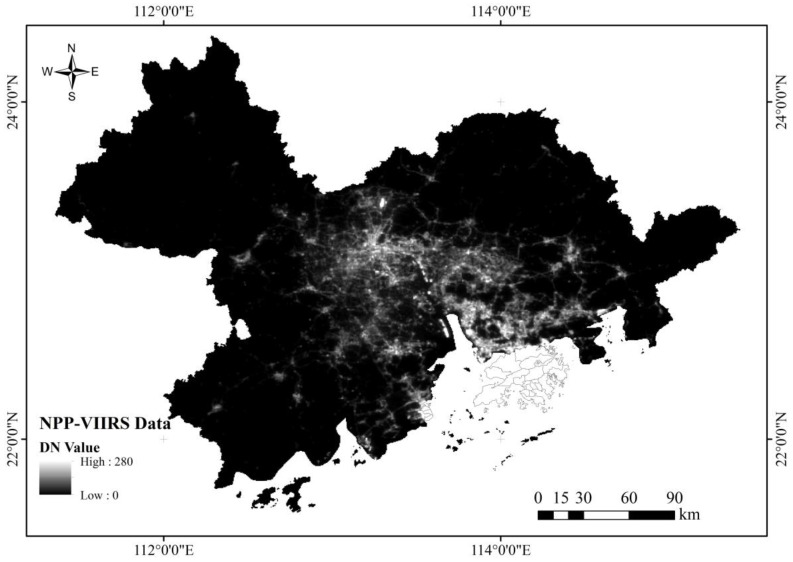
NPP-VIIRS nighttime light data of the Pearl River Delta in 2017.

The data included NPP-VIIRS nighttime lighting data (Figure 2), CO_2_ emission data from the Chinese carbon accounting database, and district and county administrative division vector data. The NPP-VIIRS nighttime lighting data were obtained from the National Geophysical Data Center (NGDC; https://eogdata.mines.edu/products/vnl; accessed on 24 May 2022). The nighttime lighting data product was 2017 composite data with a spatial resolution of 530 m from the NPP-VIIRS data source. The CO_2_ emission data at the county level for the region in the Chinese carbon accounting database [18] for 2017 are expressed in millions of tons of CO_2_. To facilitate the subsequent numerical processing, the unit was converted into tons. The vector data of administrative divisions at the district and county levels were based on data of the national administrative division at the county level at a 1:1 million scale from national basic geographic databases, which, according to the Ministry of Civil Affairs of China, reflect “the changes in administrative divisions at and above the county level of the People’s Republic of China in 2021” and thus have a good trend to strictly adjust the time scale of the data.

## 3. Methods

### 3.1. Data Preprocessing

The NPP-VIIRS nighttime lighting data used in this study were obtained from the open data source (https://eogdata.mines.edu/products/vnl; accessed on 24 May 2022) of the Earth Observation Organization, which uses the 2017 composite data. For the original NPP-VIIRS data, after initial filtering, the pixels of sunlight, moonlight, and cloud were removed, and background noise such as fire and gas combustion was not completely filtered. Additionally, there are some problems, such as negative and extreme values. The original projection coordinate system of the NPP-VIIRS nighttime light image was transformed into the Lambert equiangular azimuth projection coordinate system, and the resampling was performed at 500 m × 500 m. Then, the nighttime light image of the Pearl River Delta region was cropped using the administrative division vector data of the Pearl River Delta, and the background noise and extreme bright values were removed. (1) The background noise was filtered by selecting multiple sampling points in the rivers, lakes, and other large waters in the study area. The average value of the pixel at the sampling point was selected as the minimum light threshold, and pixels less than this threshold in the study area were assigned a value of zero. (2) Extreme brightness was filtered out; the largest pixel values of international airports (Shenzhen Bao’an International Airport and Guangzhou Baiyun Airport) in the study area were selected as the maximum lighting threshold, and pixels larger than this threshold in the study area were assigned as the maximum lighting threshold.

### 3.2. Construction of the Pixel-Scale Regression Model

#### 3.2.1. Nighttime Light Index

In this study, we used preprocessed NPP-VIIRS nighttime light data to establish a correlation between district- and county-level CO_2_ emission data. Because the minimum granularity of nighttime light data is 500 m pixels, and the scale of the original carbon emission data is at the district and county levels, the nighttime light data were aggregated by district and county, and two indicators commonly used for nighttime light data, total nighttime light (TNL) and average nighttime light (ANL), were constructed and used to characterize the nighttime lighting characteristics of the study area. It is generally believed that the larger the index is, the more intense the nighttime economic, social, and production activities are. The specific calculation method is as follows.

(1) $$\mathrm{TNL}=\overset{n}{\underset{i=1}{\sum }}{\mathrm{DN}}_{i}$$

(2) $$\mathrm{ANL}=\frac{\sum_{i=1}^{n}{\mathrm{DN}}_{i}}{n}\;\;$$

where ${\mathrm{DN}}_{i}$ represents the luminance value of pixels in the region, and n represents the number of pixels in the region.

#### 3.2.2. Correlation Analysis and Stratified Random Sampling

The Pearson correlation coefficient is widely used to measure the degree of correlation between two variables. The expression is as follows:

(3) $$r=\frac{\sum_{i=1}^{n}\left(X_{i}-\bar{X}\right)\left(Y_{i}-\bar{Y}\right)}{\sqrt{\sum_{i=1}^{n}{\left(X_{i}-\bar{X}\right)}^{2}}\sqrt{\sum_{i=1}^{n}{\left(Y_{i}-\bar{Y}\right)}^{2}}}$$

where r represents the Pearson correlation coefficient, n represents the sample size, $X_{i}$ represents the nighttime light index of sample i, $\;\bar{X}$ represents the average nighttime light index of all samples, $Y_{i}$ represents the CO_2_ emissions of sample i, and $\bar{Y}$ represents the average CO_2_ emissions of all samples. Pearson’s correlation analysis was performed for TNL, ANL, and CO_2_ emissions.

In addition, considering that the carbon emission levels of 50 districts and counties among the nine prefecture-level cities in the Pearl River Delta areas were objectively very different and had strong spatial heterogeneity, stratified random sampling was required. Stratified random sampling is a sampling method that divides the data population into several smaller and homogeneous subgroups and then conducts random sampling in the subgroups. Eighty percent of the subgroups were randomly selected for model construction.

### 3.3. Result Correction and Accuracy Test

For the regression results of CO_2_ emissions, a correction method was used for the districts and counties in the Pearl River Delta area to construct their correction coefficients, and each pixel of the regression was adjusted such that all pixels contained in each district and county were generally close to each other. Thus, CO_2_ emissions at a pixel scale of 500 square meters in the entire Pearl River Delta region were obtained after correction. The correction formula is as follows:

(4) $$\{\begin{array}{c} y_{\mathrm{ji}}^{\prime }={\;\hat{y}}_{\mathrm{ji}}\times C_{j}\\ C_{j}=\frac{Y_{j}}{{\;\hat{Y}}_{j}}\\ {\;\hat{Y}}_{j}=\sum {\hat{y}}_{\mathrm{ji}} \end{array}$$

where $y_{\mathrm{ji}}^{\prime }\;$represents the corrected CO_2_ emission of the first pixel in administrative unit j, ${\hat{y}}_{\mathrm{ji}}\;$represents the CO_2_ emission of pixel i in administrative unit j, obtained by regression, $C_{j}$ represents the correction coefficient of administrative unit j, $Y_{j}$ represents the CO_2_ emission of administrative unit j, and ${\;\hat{Y}}_{j}$ represents the CO_2_ emission of administrative unit j, obtained by regression.

Because the CO_2_ emission at the county level in the Pearl River Delta area is an observation value that is as close to the true value as possible, the root mean square error (RMSE) was used to test the accuracy. The calculation formula is as follows:

(5) $$\mathrm{RMSE}=\sqrt{\frac{\sum_{i=1}^{n}{\left({\hat{Y}}_{j}-Y_{j}\right)}^{2}}{n}}\;$$

where n represents the number of counties in the Pearl River Delta region in the verification set, which accounted for 20% of the total data, $Y_{j}$ represents the CO_2_ emissions of administrative unit j, and ${\;\hat{Y}}_{j}$ represents the CO_2_ emissions of administrative unit j, obtained by regression.

## 4. Results

### 4.1. Construction of the Optimized CO_2_ Pixel-Scale Regression Model

The two nighttime light indices of TNL and ANL and CO_2_ emissions data were used in the Pearson correlation analysis (Table 1). The TNL was significantly correlated with carbon emissions, with a correlation coefficient of 0.95, whereas no correlation was found between ANL and carbon emissions (0.039 or close to 0). Therefore, TNL was selected as the independent variable for model construction.

**Figure 3:**
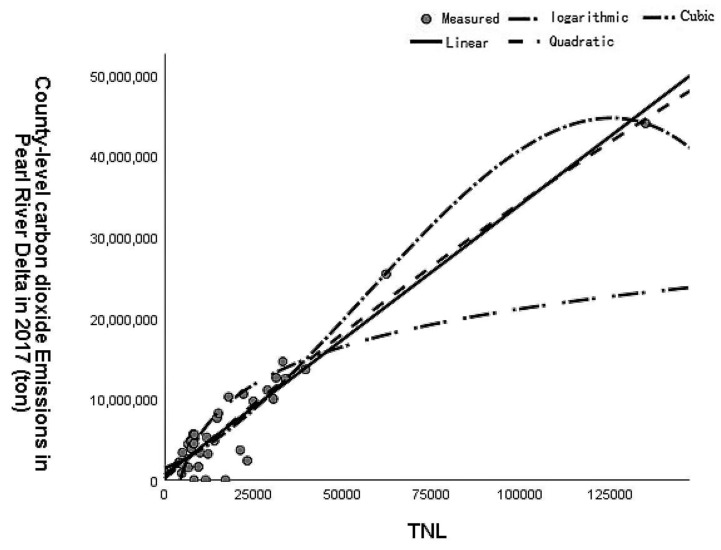
Fitting curve of total nighttime light (TNL) and CO_2_ emission model.

The results of the constructed TNL and CO_2_ emission models in Pearl River Delta counties are shown in Figure 3 and Table 2. The cubic polynomial model had the highest goodness of fit (R^2^ = 0.919), whereas the logarithmic model had the worst (R^2^ = 0.596). In addition, the linear and quadratic polynomial models also showed a high goodness of fit, which reveals that the classical polynomial model has a good fit for TNL and CO_2_.

Based on the above analysis, the cubic term model was selected to regress the pixel-scale CO_2_ emissions of the Pearl River Delta area. After correction, the RMSE of the model was 41,140.3 tons of CO_2_. Considering that the CO_2_ emissions of all districts and counties in the Pearl River Delta area are more than one million tons, the RMSE is much smaller than the magnitude of the data background, which meets the error scale of 1/10,000.

### 4.2. Spatial Distribution Pattern of Pixel-Scale CO_2_ Emissions in the Pearl River Delta

**Figure 4:**
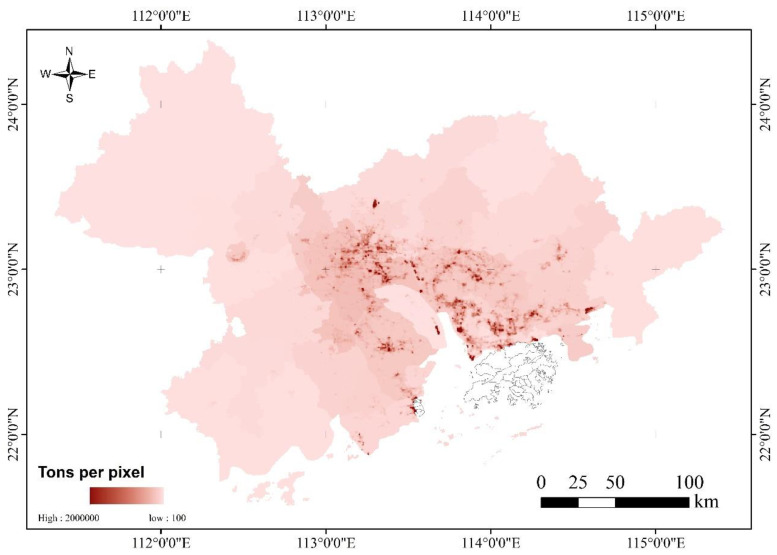
Forecast of per-pixel CO_2_ emissions in the Pearl River Delta in 2017.

Overall, the spatial distribution of CO_2_ emissions in the Pearl River Delta showed high values in a few areas, whereas CO_2_ emissions in most areas were at a low level, reflecting a considerable “center-edge” effect with distinct spatial heterogeneity (Figure 4). From the perspective of spatial distribution, Guangzhou, Shenzhen, Dongguan, Foshan, Huizhou, Zhuhai, and Zhongshan had high CO_2_ emission areas. Among them, Guangzhou, Shenzhen, Dongguan, and Foshan had more high-value regional distribution, which is consistent with their urban status; they are all cities with intense social and economic production activities, a high population attraction, and a high density of human activities. These results further confirm the validity of the conclusion that nighttime light data can be used to characterize the intensity of human activity [14,19,20,21,22,23]. The result also reveals the close relationship between CO_2_ emission levels and city status. In addition, the CO_2_ emission levels of the Zhaoqing, Jiangmen, Huizhou, Zhuhai, and Zhongshan cities, far from the center of the Pearl River Delta, were relatively low. Among them, Zhongshan and Huizhou still had sporadic high-value regional distributions, whereas Zhuhai, Zhaoqing, and Jiangmen lacked high-value areas of CO_2_ emissions, and all districts and counties in the city showed low CO_2_ emission levels. In terms of numerical distribution, the number of pixels of the first four levels of CO_2_ emissions (>2000 tons/ppx) only accounted for 20% of the entire Pearl River Delta, with the highest level accounting for only 0.28% of the total and the lowest level accounting for 80%, as shown in Table 3, indicating that the carbon emission level of most regions in the Pearl River Delta is low. Extremely high carbon emissions existed in only a small part of the region, which have a complex and far-reaching impact on the carbon emissions of the entire Pearl River Delta region.

**Figure 5:**
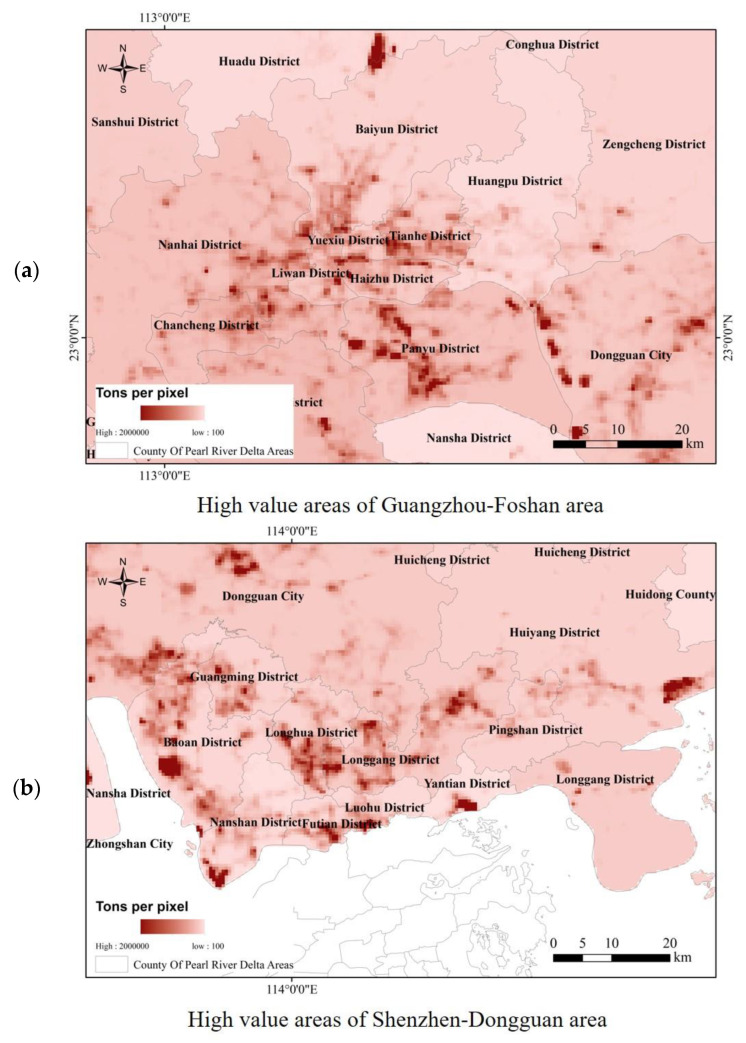
Areas with high-value CO_2_ emissions in the (**a**) Guangzhou–Foshan and (**b**) Shenzhen–Dongguan areas in 2017.

In the vicinity of high-value CO_2_ emission areas in the Pearl River Delta, there were often other high-value emission areas showing a significant “high–high” aggregation feature, and the spatial spillover effect was significant. Typical examples were the Guangzhou–Foshan and Shenzhen–Dongguan areas, as shown in Figure 5. The high-value areas of Guangzhou–Foshan were mainly located in Nanhai District, Chancheng District, and the western part of Shunde District in Foshan City and the Panyu, Haizhu, Liwan, and Tianhe districts in Guangzhou City, especially Panyu District. The high-value area presented an axial band distribution characteristic. For the Shenzhen–Dongguan area, its high-value areas were mainly located in the western, northern, and central parts of Dongguan City, as well as the Baoan, Nanshan, Longhua, Futian, Luohu, Longgang, and Yantian districts in Shenzhen. Areas with high CO_2_ emissions were distributed in almost every district-level administrative unit in Shenzhen, which is beneficial to Shenzhen’s long-term industrial development and the balanced layout of carbon sink industries. These results reflect the development of industries related to high carbon emissions in the Guangzhou–Foshan and Shenzhen–Dongguan areas. Because of the industrial characteristics of all-weather operations, they can be fully identified by the nighttime light index, which confirms the high positive correlation between nighttime light data and CO_2_ emissions.

In addition, the numerical distribution of CO_2_ emissions in Guangzhou, Foshan, Shenzhen, and Dongguan is explored, as shown in Table 4. Overall, the percentage of high-value CO_2_ emission areas of Guangzhou–Foshan and Shenzhen–Dongguan is larger than that of the Pearl River Delta as a whole (0.28%), which is 0.44% and 1.72%, respectively. In addition to the lowest carbon emission level, other levels also show this characteristic. Based on the comparison of the CO_2_ emission levels between Guangzhou–Foshan and Shenzhen City, it is found that, in the “lowest” level, the percentage of extremely low CO_2_ in Guangzhou–Foshan City is 63.06%, while that in Shenzhen and Dongguan is only 14.7%. In the “low” level, the corresponding percentage of Guangzhou Foshan City is 29.42%, while that of Dongguan and Shenzhen is 65.42%, which reveals that Dongguan and Shenzhen have more high-carbon-emission areas than Guangzhou and Foshan, while there are fewer low-carbon-emission areas.

Overall, the results are mutually verified with other similar literature, showing significant similarity in the results [24]. However, they are superior to similar studies on carbon emissions on a fined-spatial scale. Meanwhile, the research results further confirm that the overall level of carbon emissions in the Pearl River Delta region does increase with time [25].

## 5. Discussion

In this study, the NPP-VIIRS nighttime lighting data and open district- and county-scale Pearl River Delta carbon emission data were applied to construct an optimized pixel-scale regression model to predict the pixel-scale carbon emissions of Guangdong Province in 2017. From the perspective of the data source, NPP-VIIRS nighttime lighting data have great data accessibility, data quality, and temporal continuity, which proves this study could be replicated in similar situations. Compared to other nighttime lighting data sources—for example, the LJ1-01 data [17] and the DMSP-OLS data [26]—the former are only disclosed until 2018, while the latter are relatively old. However, NPP-VIIRS data have been updated and maintained all the time, although the spatial resolution is inferior to that of the Luojia-1 data. Different regression models were compared in this study, and the model was optimized by the open district- and county-scale Pearl River Delta carbon emission data. The spatial distribution pattern of pixel-scale CO_2_ emissions in the Pearl River Delta thus obtained was analyzed, which compensates for the shortcomings of traditional data, such as spatial distortion, large granularity, and insignificant spatial distribution patterns.

The main discussions are as follows.

(1) The optimized pixel-scale regression model constructed based on the NPP-VIIRS nighttime lighting data could provide fine-scale estimates of the CO_2_ emission at a pixel scale of 500 m. This study used nighttime light data to construct a nighttime light index. Several regression models were constructed through index correlation analysis and stratified random sampling, and the advantages of each regression model were compared. Cubic polynomials were selected to regress CO_2_ emissions at the pixel scale. After correction, the RMSE could reach the 1/10,000 level, and the CO_2_ emissions at the 500 m pixel scale could be estimated and predicted at a fine scale.

(2) The spatial pattern of CO_2_ emissions in the Pearl River Delta showed a distinct “center-edge” effect, with significant spatial heterogeneity. There were several high-value CO_2_ emission areas in the central and southern parts of the Pearl River Delta, whereas the eastern, western, and northern regions had low CO_2_ emissions, which reveals that there is a close relationship between CO_2_ emission levels and city status. At the numerical level, the majority of regions had low levels of carbon emissions, with only a very small number of regions having very high carbon emissions, and a small number of high-emission regions had a significant impact on the carbon emissions of the entire Pearl River Delta region.

(3) CO_2_ emissions from the Pearl River Delta showed significant spatial spillover effects and high-value aggregation. Typical areas such as the Guangzhou–Foshan and Shenzhen–Dongguan areas, corresponding to high-value areas, have developed high-carbon emission-related industries that operate around the clock, which confirms the high positive correlation between nighttime lighting data and CO_2_ emissions. At the numerical level, Dongguan and Shenzhen had a higher proportion of high carbon emissions than Guangzhou and Foshan did. In the future, when formulating carbon emission-related policies for the four core cities of the Pearl River Delta (Guangzhou, Foshan, Dongguan, and Shenzhen), more attention should be paid to Dongguan and Shenzhen to control the impact of the expansion of their high-carbon-emission areas.

## 6. Conclusions

Nighttime light data provide new perspectives and methodological tools for characterizing surface human activity-related indicators such as near-surface CO_2_ emissions [20,27,28,29,30,31,32,33,34]. In this study, the NPP-VIIRS data source was used to determine CO_2_ emissions at the pixel scale in the Pearl River Delta area in 2017. Compared to the traditional top-down or bottom-up method [35,36], this study used the NPP-VIIRS data source [13] with CO_2_ emissions at the district and county levels in China to construct a quantitative model to project CO_2_ emissions at the 500 m image metric scale in the Pearl Delta River area for 2017 to estimate and explore the spatial distribution pattern. The results of the study confirm the interpretability of nighttime light data in the perception of CO_2_ emissions, revealing that the CO_2_ emissions in the Pearl River Delta region show significant spatial heterogeneity and high-value aggregation characteristics. However, this study did not consider a richer time-and-space scale and only focused on the single time node of 2017. In the future, it will be possible to introduce more remote sensing big data of different scales and even multi-source heterogeneous big data for fusion to construct a multi-time series analysis of different time sections, or even high-time-resolution and more fine-scale CO_2_ emission prediction research for use in policy formulation. Fine-scale carbon emission research can be introduced as a consideration in various forums, such as public decision making and social distribution [37], to provide a reference for the realization of China’s long-term goals.

## Figures and Tables

**Table 1:** Correlation analysis between the nighttime light index and CO_2_ emissions. TNL, total nighttime light; ANL, average nighttime light.

	TNL	ANL
r (Pearson)	0.950	−0.039
Significance	0.000	0.787

**Table 2:** Comparison of regression results.

Model Summary				Parameter Estimated Value			
Model	R^2^	F	Significance	Constant	b1	b2	b3
Linear	0.909	380.551	0.000	647,055.855	334.133		
Logarithm	0.596	56.157	0.000	−56,557,734.916	6,748,511.249		
Quadratic	0.911	189.890	0.000	192,425.593	374.718	0.000	
Cubic	0.919	136.401	0.000	1,479,980.790	171.999	0.006	−3.293 × 10^−8^

**Table 3:** Proportion of regions with different CO_2_ emissions.

CO_2_ Emission Level	Area (m^2^)	Number of Pixels	Percentage (%)
High (>20,000 tons/ppx)	156,500,000	626	0.28
Medium (10,000–20,000 tons/ppx)	336,250,000	1345	0.61
Relatively Low (5000–10,000 tons/ppx)	1,580,750,000	6323	2.85
Low (2000–5000 tons/ppx)	9,016,500,000	36,066	16.24
Very Low (<2000 tons/ppx)	44,434,250,000	177,737	80.02

**Table 4:** Proportion of different levels of CO_2_ emissions in the Guangzhou–Foshan and Shenzhen–Dongguan areas.

CO_2_ Emission Level	Guangzhou–Foshan			Shenzhen–Dongguan		
	Area (m^2^)	Number of Pixels	Percentage (%)	Area (m^2^)	Number of Pixels	Percentage (%)
High (>20,000 tons/ppx)	97,500	195	0.44	158,000	316	1.72
Medium (10,000–20,000 tons/ppx)	246,000	492	1.1	336,500	673	3.67
Relatively Low (5000–10,000 tons/ppx)	1,335,500	2671	5.98	1,329,500	2659	14.49
Low (2000–5000 tons/ppx)	6,574,000	13,148	29.42	6,002,000	12,004	65.42
Very Low (<2000 tons/ppx)	14,094,000	28,188	63.06	1,349,000	2698	14.7

## Data Availability

The data presented in this study are available from the authors upon reasonable request.
